# Computational approaches to predicting treatment response to obesity using neuroimaging

**DOI:** 10.1007/s11154-021-09701-w

**Published:** 2021-12-23

**Authors:** Leonard Kozarzewski, Lukas Maurer, Anja Mähler, Joachim Spranger, Martin Weygandt

**Affiliations:** 1 Charité – Universitätsmedizin Berlin, corporate member of Freie Universität Berlin and Humboldt-Universität zu Berlin, Clinic of Endocrinology, Diabetes and Metabolism, 10117 Berlin, Germany; 2 Charité – Universitätsmedizin Berlin, corporate member of Freie Universität Berlin and Humboldt-Universität zu Berlin, Charité Center for Cardiovascular Research, 10117 Berlin, Germany; 3 Berlin Institute of Health at Charité – Universitätsmedizin Berlin, 10117 Berlin, Germany; 4 Max Delbrück Center for Molecular Medicine and Charité – Universitätsmedizin Berlin, corporate member of Freie Universität Berlin and Humboldt-Universität zu Berlin, Experimental and Clinical Research Center (ECRC), 13125 Berlin, Germany; 5 DZHK (German Center for Cardiovascular Research), partner site Berlin, Berlin, Germany; 6 Charité – Universitätsmedizin Berlin, corporate member of Freie Universität Berlin and Humboldt-Universität zu Berlin, NeuroCure Clinical Research Center, 10117 Berlin, Germany

**Keywords:** Personalized medicine, Obesity treatment, Machine learning, Task-fMRI, Resting-state fMRI, Biomarkers

## Abstract

Obesity is a worldwide disease associated with multiple severe adverse consequences and comorbid conditions. While an increased body weight is the defining feature in obesity, etiologies, clinical phenotypes and treatment responses vary between patients. These variations can be observed within individual treatment options which comprise lifestyle interventions, pharmacological treatment, and bariatric surgery. Bariatric surgery can be regarded as the most effective treatment method. However, long-term weight regain is comparably frequent even for this treatment and its application is not without risk. A prognostic tool that would help predict the effectivity of the individual treatment methods in the long term would be essential in a personalized medicine approach. In line with this objective, an increasing number of studies have combined neuroimaging and computational modeling to predict treatment outcome in obesity. In our review, we begin by outlining the central nervous mechanisms measured with neuroimaging in these studies. The mechanisms are primarily related to reward-processing and include “incentive salience” and psychobehavioral control. We then present the diverse neuroimaging methods and computational prediction techniques applied. The studies included in this review provide consistent support for the importance of incentive salience and psychobehavioral control for treatment outcome in obesity. Nevertheless, further studies comprising larger sample sizes and rigorous validation processes are necessary to answer the question of whether or not the approach is sufficiently accurate for clinical real-world application.

## Introduction

The global obesity epidemic is one of today’s major public health concerns. According to the World Health Organization, 650 million adults or 13% of the world’s adult population were obese in 2016 and the worldwide prevalence in adults nearly tripled between 1975 and 2016. In addition to these concerning epidemiological characteristics, obesity is associated with multiple adverse consequences, including increased risk for cardiovascular disease, diabetes, cancer, premature mortality as well as depression and anxiety [1–7]. Besides social [8], genetic [9], hormonal [10] and behavioral [11–13] factors, central nervous factors promote the development and maintenance of obesity. Neural mechanisms that (i) underlie impaired food reward [14, 15], (ii) link food cues to (anticipated) reward [16, 17] and (iii) underlie reduced psychobehavioral control [16, 18, 19] are considered the main central nervous contributors to obesity. In developed countries these effects are increased by 24-h advertisement and availability of low cost, calorie dense, and highly palatable food.

Compatible with this multi-factorial etiology, three groups of treatments exist: lifestyle interventions (LIs), bariatric surgery (BS) and pharmacological interventions (PIs). LIs include caloric restriction, physical exercise, eating behavior modification and dietary counselling. Balanced hypocaloric diets induce clinically meaningful weight loss [20]. Optimal weight loss and maintenance are achieved when caloric restriction and physical exercise are combined [21, 22]. However, long-term weight regain is relatively common [23].

BS is currently the most effective treatment with regard to weight loss, attenuation of comorbidity (e.g., type 2 diabetes) and mortality prevention [24, 25] and thus the treatment of choice for severe obesity [26]. Sleeve gastrectomy (stomach volume reduction to 80 to 120 mL) is the most frequently recommended BS technique. Nevertheless, there is considerable variability in weight loss. Weight regain occurs in 20–30% of patients [27–34]. Long-term weight regain has been associated with a reversal of surgery-induced hormonal variations (e.g., in ghrelin and GLP-1; [35]), post-bariatric hypoglycemia [36], dietary non-adherence [37], and physical inactivity [38]. Problematic behavioral patterns are likely further aggravated in psychiatric patients [39]. The safety of BS has improved drastically in the last two decades, with perioperative mortality rates ranging from 0.03 to 0.2% [40–43]. However, complications can still occur and include early complications such as bleeding, thromboembolism, bowel obstruction and wound infection and as well as late complications such as stricture, reflux disease, cholelithiasis, hernia, nutritional and vitamin deficiencies, and dumping syndrome [44, 45]. In the long term up to 22% of patients require reoperation [46–49]. Due to the differences in efficiency and risk between treatment options, a prognostic tool which predicts treatment success and could thus guide individual treatment choices in a personalized medicine framework is highly desirable.

Only few drugs are used in clinical obesity management, including Orlistat, a pancreatic lipase inhibitor, Phentermine/topiramate, a sympathomimetic, appetite suppressant, Lorcaserin, a 5-HT2C receptor activator, Naltrexone/bupropion, a transmitter reuptake inhibitor and Liraglutide, a glucagon-like peptide 1 (GLP-1) analogue. Especially GLP1-analoge trials have produced promising results with Liraglutide treatment usually resulting in a weight loss of 4 to 6 kg and Semaglutide treatment demonstrating even greater weight loss [50, 51]. Side effects of liraglutide include gastrointestinal symptoms, such as nausea, diarrhea, constipation, and vomiting [52]. PI weight loss is partly or completely reversed after treatment [53]. PIs are only recommended as add-on to LIs [54]. See [54] for a more detailed overview on LI, [55] for BS, and [52] for PI.

Currently, a variety of studies exist that use computational approaches and neuroimaging signals to predict treatment outcome in obesity. In this review, we explain the key central nervous mechanisms assessed in these studies, present the different neuroimaging and computational prediction techniques, and give a detailed overview of existing studies. In the Discussion, we pay special attention to the questions of (i) whether the results obtained are sufficient to legitimate clinical real-world applications (which is presumably not yet the case), (ii) what could be done to meet this requirement and (iii) how statistical analyses could be improved to provide more detailed models for “treatment” and “treatment outcome”.

We included longitudinal LI, BS, and PI studies. We required a period of at least one months between treatment initiation and the latest follow-up. Compatible with the general meaning of “prediction” as a forecast of future events and of “prediction” as a statistical process modelling some factor based on other factors, we included studies that prognose future treatment outcome using neuroimaging biomarkers and studies that predict treatment-induced variations in outcome markers based on variations in neuroimaging parameters across treatment.

## Central nervous mechanisms affecting body weight

At least partially motivated by the discovery of overlapping psychobehavioral symptoms in persons with obesity and substance dependence such as loss of control over consumption and craving [16, 56], neuroimaging research on central nervous parameters impacting body weight has focused on three major reward-related mechanisms: Reward system hyposensitivity to food consumption, reward system hyperresponsivity to stimuli predicting food consumption, and dysfunctional psychobehavioral or goal-directed control system respectively.

### Reward system hyposensitivity to food consumption

A reduced sensitivity of the brain reward system to food consumption (including the actual pleasurable impact of consumption) is regarded as a factor that triggers excessive overeating as a method of compensation [14, 15, 57]. In accordance with addiction research, a reduced striatal dopamine (DA) release after food consumption and reduced availability of DA receptor subtype D2 (D2R) are discussed as causes for said hyposensitivity.

Specifically, Small et al. found a food-intake induced DA release after a 16 h fast that reflected the pleasantness of food consumption in normal weight persons [58]. This is compatible with findings showing that amphetamine-induced DA release correlates with the experienced pleasantness of amphetamine consumption in healthy subjects [59] and a reduced DA release in detoxified cocaine abusers [60]. The pleasurable impact of consumption is frequently referred to as “liking” [17]. Van de Giessen et al. found reduced DA release in obese persons in the sense that amphetamine induced a significant DA release in lean but not obese persons [57]. Based on these findings, it was concluded that blunted DA release after consumption is a mechanism underlying compensatory overeating in obesity (e.g., [57]). Moreover, in agreement with findings made for addiction [61], obesity research found that the availability of striatal D2R is significantly decreased in obese persons [15, 62] and is negatively related to their BMI [15]. Consistently, Johnson & Kenny found a progressive decrease in D2R-availability accompanied by a progressive increase in compulsion-like overeating in rats randomized into an overeating condition compared to control animals [14] and Geiger et al. found reduced extracellular dopamine in the striatum in a comparable experimental setting [63]. Johnson & Kenny concluded that the observed progressive dopaminergic hyposensitivity reflects the transition from normal to compulsive overconsumption [14].

However, some findings question the importance of (dopaminergic) reward system hyposensitivity as a risk factor for weight gain. Hardman et al. found that suppressing DA signals leads to a reduced food intake in humans [64]. Tellez and colleagues showed that down-regulation of DA release due to a prolonged high-fat diet reduces caloric intake in rodents [65]. Several studies on addiction research depleting/antagonizing DA functioning did not find an impact on drug or food-liking. Only a reduction in motivational properties (i.e., of “drug-wanting”, see Sect. 2.2) was found [66, 67]. Importantly, Tellez and colleagues also showed that application of oleoylethanolamine restores DA release [65]. Oleoylethanolamine is a lipid messenger whose synthesis is suppressed due to the high-fat diet. Their study provides a mechanism that explains reduced DA release as a consequence of a prolonged high-fat diet. Consistent with this description of reduced DA release as a consequence (and not a cause) of an excessive calorie intake and given the experimental design applied by Johnson & Kenny [14], one might assume that the progressive decrease in D2R-availability observed in their study reflects the end-point of rather than the transition to compulsive overconsumption. See [68–70] for an overview. Finally, the exact role of calorie-intake related DA receptor alterations appears unclear due to findings of Dobbs et al. showing that D2R downregulation can be associated with a D1R hyper-reactivity, suggesting more heterogeneous DA adaptations [71].

Therefore, a causative role of a DA-mediated reward system hyposensitivity for weight gain remains debatable. Only one of the two Positron-Emission-Tomography (PET) studies directly evaluating a link between DA functioning and future weight loss [72, 73] found such a link [72]. Authors elaborating on alternative neurotransmitters suggest endorphins or endocannabinoids as substrates of drug or food liking (see e.g., [17] for an overview). An important role of endorphins would be consistent with findings of the PET study not showing a link for DA [73] but showing negative associations between body weight variations and μ-opioid receptor availability in amygdala, insula, ventral striatum, and putamen. In conclusion, additional research on DA-based reward hyposensitivity appears necessary given contradictory findings.

### Reward system hyperresponsivity to stimuli predicting food consumption

“Incentive salience” is a motivational mechanism considered to initiate compulsive food seeking and consumption after food cue exposure because these cues were coupled to reward (consumption) by Pavlovian conditioning in the learning history of an individual. Consequently, food cues are predictive of food intake and can acquire similar motivational properties as food reward after repeated couplings [74]. Incentive salience relies on a hypersensitivity of the DA reward system to these cues [17] and corresponds to a strong desire, a strong “I want to consume feeling” on a psychological level. This desire has consistently been termed as “wanting” [17] or “craving” [56].

Early work relating this cue-dependent motivational mechanism to striatal DA was done by Schultz et al. who showed in a pivotal animal study that the response of striatal DA neurons varies across different stages of food exposure. Animals respond to palatable food consumption during early stages of exposure, but only to cues predicting consumption after repeated exposure [75]. Hamid et al. linked striatal DA to cue-sensitivity by showing that it reflects the willingness to engage in effortful activities to obtain reward after cue exposure in rats [76]. Furthermore, the role of striatal DA for food wanting in humans was underlined by van de Giessen et al. who found that DA release after amphetamine intake in obese persons correlated with food craving on trait level [57].

Although incentive salience is primarily a motivational phenomenon, it also comprises attentional, affective, learning-related, and behavioral facets. Consistently, functional magnetic resonance imaging (fMRI) studies using cue reactivity (CR) tasks did not only find striatal hyperresponsivity to high-calorie food cues but also a hyperresponsivity in anterior cingulate cortex and visual areas, amygdala, orbitofrontal cortex, and hippocampus [77–80]. CR tasks represent the key functional paradigms for studying incentive salience which contrast neural signals emerging during perception of food cues to those during control conditions (see 3.1.1). In this framework, the anterior cingulate cortex / amygdala / visual areas are supposed to modulate the attentional [81] / emotional [82] / sensory salience of food cues (cf. [83]). The orbitofrontal cortex might underlie stimulus – outcome encoding in Pavlovian conditioning [84]. The hippocampus plays an inhibitory role in appetitive Pavlovian conditioning [82]. Consistent with the concept of incentive salience, a hyperresponsivity in these areas was found to predict unfavorable treatment outcome in a variety of reviewed CR studies.

However, some studies did not support a link between incentive salience and treatment outcome. Specifically, neither the BS fMRI study of Bach et al. nor the PI fMRI study of Ten Kulve et al. found significant associations between brain activity evoked by food-cue presentation before the treatment and treatment-induced weight loss [85, 86].

### Dysfunctional goal-directed control system

A dysfunctional psychobehavioral or goal-directed control system and reduced modulation of incentive salience by this system is considered a further mechanism contributing to overeating [19]. This can be understood when viewing eating behavior from a decision-making perspective. The Pavlovian incentive salience mechanism primarily mediated by the striatal DA system can be seen as a decision-making mechanism favoring choices that have previously been associated with immediate and highly rewarding consequences. In line with its subcortical location, this striatal mechanism does not consider future consequences [19]. By contrast, the goal-directed decision-making system is driving (food) choices by comparing different options based on action plans encoding their present and future consequences [19, 87]. Thus, this system could inhibit the impulse to eat a tasty but unhealthy food (e.g., triggered by the striatal DA system) because it predicts that the negative consequences of future overweight outweigh (i.e., have a higher negative value) the positive consequences of immediate reward (i.e., their positive value) [19].

Hare et al. identified value-based goal-directed decision-making regions in the brain by having self-reported dieters choose between two food items: a constant reference item with average taste- and health related properties and another that varied in these aspects [88]. Ventromedial prefrontal cortex (vmPFC) activity predicted the food choice (i.e., its value) independent of the food’s tastiness or healthiness. Activity in the dorsolateral prefrontal cortex (dlPFC) reflected self-control (i.e., was higher when subjects chose healthy). VmPFC and dlPFC activity correlated only during successful self-control trials. The authors concluded that the vmPFC computes a value-signal which determines food-choice and relies on both factors, reward (taste) and control (health) only when it reflects control-related dlPFC activity. VmPFC activity alone only reflects reward (taste).

Another study employed a delay discounting (DD) paradigm [87]. In DD tasks, participants have to decide repeatedly between rapidly available smaller rewards or larger rewards available at a later time (see 3.1.2). A weaker preference for earlier smaller than for larger delayed rewards is considered as a behavioral marker for goal-directed control. This study highlighted the importance of the interplay between fronto-parietal control areas and striatal incentive salience areas for goal-directed control. Stronger goal-directed control depends on stronger lateral-prefrontal relative to striatal activity. Please see [89] for findings suggesting an inhibitory impact of prefrontal on incentive salience regions including striatal ones (modulated by the specific calorie-restriction type applied). A direct link between key regions of goal-directed and striatal Pavlovian control is consistent with the finding that DA depleted mice do not at all initiate goal-directed behaviors including feeding [90]. In addition, animal studies suggest that the insular cortex also contributes to goal-directed decision-making as lesions to this area impaired the ability of rats to devalue food after satiety and to adjust their food choice accordingly [91].

The clinical importance of this factor has been demonstrated on a behavioral level in DD studies showing reduced goal-directed control in obese persons [92, 93]. These studies controlled for nuisance factors (e.g., age and income). Studies not controlling for these variables failed to show these effects (e.g., [94, 95]). Neuroimaging studies in obese subjects revealed a link between reduced D2R availability in the striatum and a reduced resting-state (RS) glucose metabolism in regions involved in goal-directed decision-making such as vmPFC and dlPFC [96]. In [97] we could demonstrate the importance of behavioral and neural measures of goal-directed control and their interplay with striatal Pavlovian regions for the dietary success of obese persons in a 12-week LI. Higher behavioral goal-directed control was coupled to better weight loss. Functional connectivity (FC) between vmPFC and dlPFC was positively related to behavioral control and weight loss and FC between vmPFC and dorsal striatum was negatively linked with future weight loss. We evaluated the role of the interplay between Pavlovian and goal-directed neural systems in a LI study by testing whether future dietary weight loss and long-term maintenance after treatment across 39 months could be predicted based on activity assessed in a food CR paradigm, a food-specific DD paradigm, and the interaction of these activities [18]. This revealed a strong link between future long-term weight loss and interactions between visual Pavlovian and insular control areas.

## Neuroimaging techniques and parameters used for prediction

**Fig. 1:**
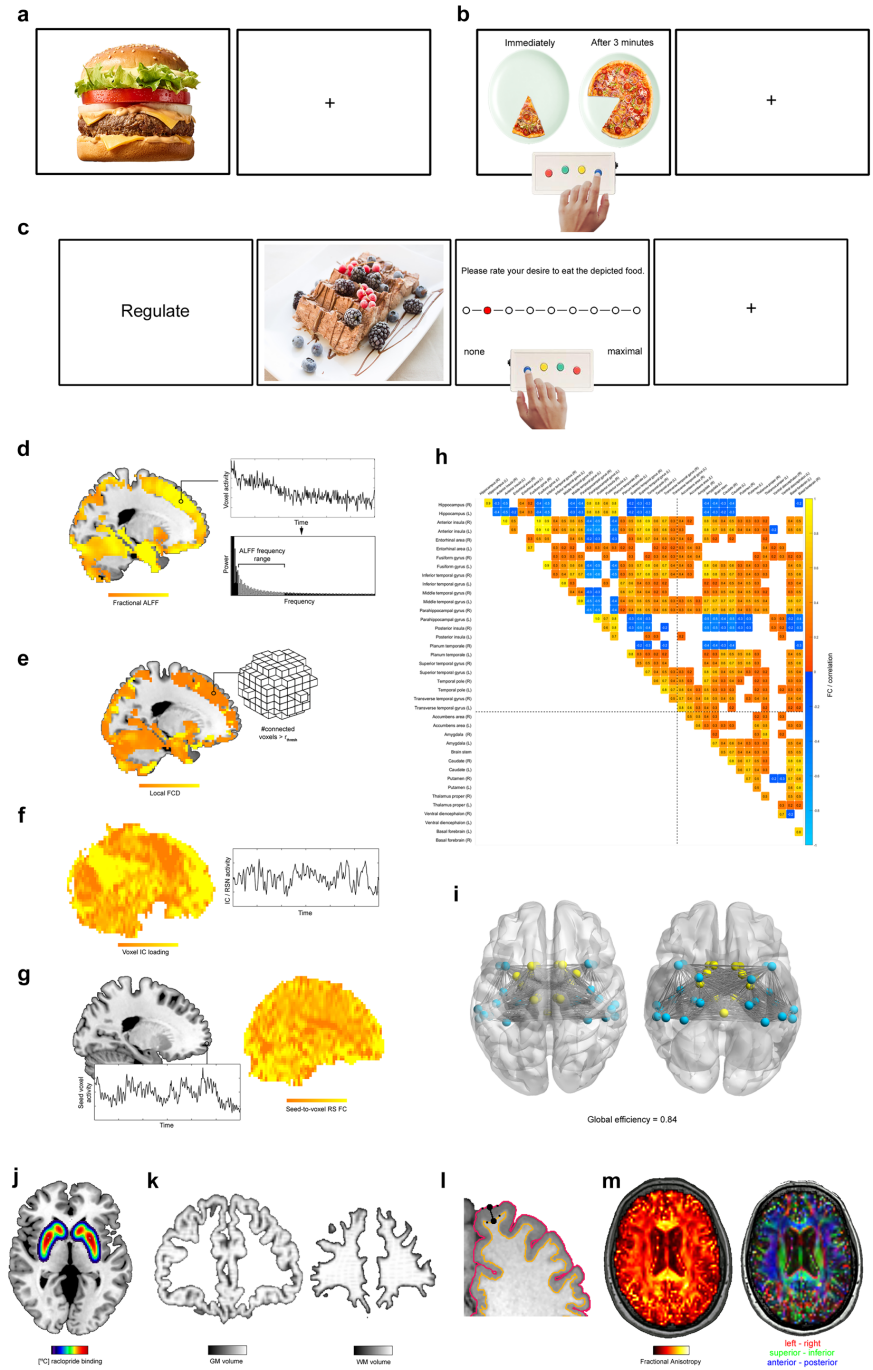
Neuroimaging techniques and parameters utilized in the reviewed studies. (**a**)–(**c**) illustrate the basic layouts of the three fMRI tasks, i.e., CR (**a**), DD (**b**), and food CrvR (**c**). The panels (**d**)–(**i**) depict the different parameters derived from RS fMRI. In particular, 1d illustrates the ALFF method, (**e**) FCD mapping. (**f**) shows a component loading map for a RS-network extracted by independent component analysis. Moreover, (**g**) illustrates the seed-to-voxel FC approach. (**h**) shows a correlation (i.e., FC) matrix obtained for temporal and deep GM regions for RS fMRI data of a single subject and time point. FC depicted is thresholded at r =|0.5|. (Only) temporal and deep GM regions were selected to facilitate a better readability of the panel. The network depicted in (**i**) corresponds to the areas / FC depicted in the correlation matrix in (**h**). This network has a global efficiency of 0.84. (**j**) illustrates a PET scan using the [^11^C] raclopride radio-tracer. Finally, (**k**)–(**m**) depict the structural MRI measures. Specifically, (**k**) shows a brain voxel map of the GM (left) and WM (right) volume of a participant determined with VBM. (**l**) illustrates an approach to cortical thickness estimation that treats the distance between two closest vertices on the opposing WM/GM surface and the GM/pial surface as measure of cortical thickness for the corresponding cortex segment. (**m**) illustrates the fractional anisotropy determined with DTI for a single participant and time point on the left. In order to illustrate the directional information contained in DTI maps (and used for fiber tractography), the direction of the first tensor for a given voxel is depicted with a red–green–blue coding on the right. For further details, see text

Task- and RS-fMRI as well as structural MRI (sMRI) are the neuroimaging acquisition techniques predominantly employed in the reviewed studies. fMRI provides indirect markers of neural activity by measuring vascular responses to heightened metabolic demands of active neurons [98] while sMRI provides information on various brain tissue characteristics. Neuroimaging parameters derived for prediction from fMRI and sMRI can be subdivided in two major groups: Parameters characterizing specific, localized processing of individual brain regions (“functional segregation”) and those reflecting the interplay or FC of activity among different regions respectively (“functional integration”). All methods described in this section are illustrated in Fig. 1.

### Task-fMRI

Three task-based fMRI paradigms outlined below are currently used for treatment outcome prediction in obesity: CR, DD, and food craving regulation (CrvR). [97, 99–101] used them to derive measures of FC, the rest exclusively computed markers of localized activity in individual brain coordinates (i.e., voxels).

In these task-fMRI studies, markers of localized activity (referred to as “Voxel CR, DD, or CrvR activity” in the following), are computed in a two-step process. First, three-dimensional maps of neural activity reflecting the targeted mechanism in individual voxels are determined for each participant and time point. Second, these parameters are entered into a predictive group-level analysis utilizing methods described in Sect. 4.

Task-related FC markers are also computed in a two-step procedure (“Seed-to-voxel CR FC” or “Seed-to-voxel DD FC”) in the majority of studies evaluating task-related FC [97, 99, 100]. First, a seed coordinate sensitive to the evaluated factor is selected based on prior knowledge and the association between its time series (potentially modulated by the time course of a condition of interest [99, 100]; see [102]) and all other voxels is computed. Second, the voxel-wise correlation/regression coefficients are entered into a group-level analysis.

#### Cue reactivity

The key experimental design to study incentive salience, which is applied in the majority of all reviewed studies (see Tables 1 - 3), is the food CR paradigm. Reflecting the notion that exposure to food-cues (e.g., pictures, taste, odor, or imagined food items) can trigger food-wanting/craving and subsequently food-intake [74], CR tasks typically present pictures of high-calorie food and control items such as pictures of neutral objects or low-calorie foods. Participant-specific voxel contrast maps reflecting activity related to incentive salience are then computed by subtracting activity during the control condition from activity during presentation of palatable foods to control for non-food related activation.

**Table 1:** Overview on existing lifestyle intervention studies. Studies are subdivided by neuroimaging technique. Studies may be listed more than once if more than one neuroimaging technique is applied. In order to ease comprehensibility of this and the two other tables, we occasionally simplified the presented study characteristics. In particular, if a study comprised the prognostic and associative modelling approach, only one was mentioned (in this case the study would have been classified as “Prognostic” in the column “Modelling: Outcome” as we considered this modelling approach more meaningful). Similarly, if a study comprised several outcome markers, only one was mentioned in the column “Modelling: Outcome”. In case changes in bodyweight or weight loss respectively was modelled in addition to other outcome markers, “bodyweight” was mentioned in the column “Modelling: Outcome” as we considered this outcome most relevant. Consistently, “Predictor” and “Significant prediction results” only list those predictors and results that relate to the parameters reported in column “Modelling: Outcome”. The codes for modelling parameters and outcomes in “Significant prediction results” (such as “CR_fMRI_T0 → OUT_T3” or “PET_T0 → OUT_T3”) report the predictor and the time point(s) the predictor was derived of on the left sign of the arrow, on the right side the time points for the outcome marker are reported. Despite slight potential inaccuracies resulting from this procedure, a period of four weeks was converted to one month in these time point codes. In column “Experimental design “, these time points are reported on a (higher) week-level accuracy. Consequently, for example, the code “CR_fMRI_T0 → OUT_T3” would refer to modelling an outcome marker measured after three months of treatment based on a food CR fMRI parameter measured immediately before treatment onset. Finally, the number of time points listed after each “T” in these codes is important: if only a single time point is listed after a “T” (such as in “OUT_T0” or “OUT_T0_T3”) than parameter raw values measured at the specific time point(s) were modelled with one model – in this case for the baseline time point alone (“OUT_T0”) or the baseline time point and a time point after 3 months (“OUT_T0_T3”). If, however, a “T” is followed by two time points (such as in “OUT_T0_3_T3_9”) than temporal difference markers for the respective parameter were modelled with one model – in this case differences between baseline and after 3 months, and after 3 vs. 9 months. Thus, for example, the code „CR_fMRI_T0_T3_T15_T27 → OUT_T0_3_T3_15_T15_27_T27_39 “ refers to one model were temporal differences in an outcome marker between time points T0 & T3, T3 & T15, T15 & T27, and finally T27 & T39 were modelled on CR fMRI parameters sampled at T0, T3, T15, T27. Finally, in addition to the studies presented in the table, we want to mention an LI study of Hege et al. [195] who used magnetoencephalographic data to prognose future WL in 33 overweight or obese participants. They found that higher activity in superior temporal gyrus, fusiform gyrus, hippocampus, inferior temporal gyrus, insula, Heschl gyrus, fusiform gyrus, insula went along with successful weight loss, lower activity in middle occipital and inferior frontal gyrus went along with successful weight loss. We mention this study separately, as the measurement technique (magnetencephalography) and the task applied (1-back memory task) deviate strongly from the other studies presented in this work

Study	Modelling: Outcome	#Participants (per group)	Experimental design	Predictor	Pred. meth	Significant prediction results
Task-fMRI						
Schur et al. [173]	Prognostic: Bodyweight	37 OB (WL: Family-based behavior treatment)	T0 (pre-WL), T6 (after 6 months WL), T12 (after 6 months FUP 1 + WL), T18 (after 6 months FUP 2 + WL + FUP1)	Voxel CR activity	OLS	CR_fMRI_T0 → OUT_T0_6: Positive association between WL and the reduction in CR activity to high-calorie food cues averaged across medial orbitofrontal cortex, substantia nigra/ventral tegmental area, amygdalae, dorsal and ventral striatum, and insula from before to after consuming a standardized meal
Hermann et al. [174]	Prognostic: Bodyweight	29 OB (WL: 29 caloric restriction, nutritional counselling)	T0 (pre-WL), T1 (after 1-month WL), T6 (after 6 months WL)	Voxel CR activity	OLS	CR_fMRI_T0_1 → OUT_T0_6: Positive association between WL and CR activity reductions in posterior part of the dorsal and ventral putamen, right pallidum, and left caudate
Maurer et al. [163]	Prognostic: Bodyweight	23 OW/OB (WL: 19 caloric restriction; WMT: 11 nutritional counselling, PE, Relaxation, 12 ad lib)	T0 (pre-WL), T3 (after 12 weeks WL), T15 (after 12 month WMT + WL), T27 (after 12 months FUP + WL + WM)	Voxel CR activity (modulated by GLP1)	LMM	CR_fMRI × GLP1_T0_T3_T15 → OUT_T0_3_T3_15_T15_27: Positive association of body weight variations with the interaction of endogenous Glukagon-Like-Peptid-1 levels and CR activity in dlPFC
Neseliler et al. [179]	Prognostic: Bodyweight	24 OW/OB (WL: 24 caloric restriction)	T0 (pre-WL), T1 (after 1-month WL), T3 (after 3 months WL)	CR activity averaged across selected voxels	OLS	CR_fMRI_T0_1 → OUT_T1_3: Positive association between WL and the average CR activity of regions including control areas (dlPFC, inferior frontal gyrus, inferior parietal lobule)
Szabo-Reed et al. [169]	Prognostic: Bodyweight	75 OW/OB (WL: 75 caloric restriction, behavioral modification, physical activity)	T0 (pre-WL), T3 (after 3 months WL)	Voxel CR activity	SEM	CR_fMRI_T0 → OUT_T0_3: Positive effect of right middle frontal gyrus CR activity on WL mediated via program attendance (positive effect of right middle frontal gyrus (i.e., dlPFC) CR activity on attendance; positive effect of attendance on WL)
Weygandt et al. [18]	Prognostic: Bodyweight	30 OW/OB (WL: 22 caloric restriction; WM: 12 nutritional counselling, PE, relaxation, 18 ad lib)	T0 (pre-WL), T3 (after 12 weeks WL), T15 (after 12-month WM + WL), T27 (after 12 months FUP1 + WL + WMT), T39 (after 12 months FUP2 + WL + WMT + FUP1)	Principal components of voxel CR and DD activity	LMM	CR_fMRI_T0_T3_T15_T27 → OUT_T0_3_ T3_15_T15_27_T27_39: Moderate negative association between WL and CR network activity (esp. left hippocampus) DD_fMRI_T0_T3_T15_T27 → OUT_T0_3_ T3_15_T15_27_T27_39: Moderate positive association between WL and DD network activity (esp. right inferior parietal gyrus) CR × DD_fMRI_T0_T3_T15_T27 → OUT_T0_3_ T3_15_T15_27_T27_39: Strong positive association between WL and interaction of CR network activity (esp. visual areas) with DD network activity (esp. right insula)
Paolini et al. [101]	Prognostic: Bodyweight	56 OW/OB (WL & WMT: caloric restriction, caloric restriction + aerobic exercise, caloric restriction + resistance exercise; no group sizes provided)	T0 (pre-WL), T6 (after 6 months WL)	Global efficiency derived from CR FC	OLS	CR_fMRI_T0 → OUT_T0_6: Positive association between WL and global efficiency of a network comprising primarily reward areas
Weygandt et al. [106]	Prognostic: Bodyweight	23 OW/OB (WMT: 10 nutritional counselling, PE, Relaxation, 13 ad lib)	T0 (post-WL), T12 (post 12-month WMT)	Voxel DD activity	OLS	DD_fMRI_T0 → OUT_T0_12: Positive association between WL and superior frontal gyrus DD activity
Weygandt et al. [97]	Prognostic: Bodyweight	16 OW/OB (WL: 16 caloric restriction)	T0 (pre-WL), T3 (after 12 weeks WL)	Voxel DD activity; Seed-to-voxel DD FC	OLS	DD_fMRI_T0 → OUT_T0_3: Positive association between WL and DD activity in vmPFC, dmPFC, and dlPFC; negative association between WL and DD activity in anterior insula and dmPFC; DD_fMRI_T0 → OUT_T0_3: Positive association between WL and DD FC of vmPFC with dlPFC and dmPFC. Negative association between WL and DD FC of vmPFC with dorsal striatum
Murdaugh et al. [175]	Prognostic: Bodyweight	25 OW/OB (WL: 25 educational, motivational, and behavioral components)	T0 (pre-WL), T3 (after 12 weeks WL), T12 (after 9 months FUP + WL)	Voxel CR activity	OLS	CR_fMRI_T0 → OUT_T0_3: Negative association between WL and activity in reward areas (ncl. accumbens, ACC, frontal operculum, insula), visual processing areas, areas mediating attentional processes, middle temporal gyrus, cerebellum; CR_fMRI_T3 → OUT_T3_12: Negative association WL and CR activity in reward areas (ventral tegmental area, putamen, insula, and hippocampus), visual processing and attention areas; CR_fMRI_T0_3 → OUT_T3_12: Positive association between WL and reduction in CR activity in insula, inferior frontal gyrus, thalamus
Drummen et al. [180]	Associations: Bodyweight	27 OW/OB (WL: caloric restriction; WMT: 12 moderate protein, 15 high protein dietary guidelines)	T0 (pre-WL), T2 (after 8 weeks WL), T26 (after 24-month WMT + WL)	Voxel CR activity	OLS	CR_fMRI_T2_26 → OUT_T2_26: Negative association between changes in body weight during weight maintenance and changes in neural food CR during weight maintenance in left and right rolandic operculum, right inferior frontal gyrus, and left middle frontal gyrus (i.e., dlPFC)
Resting-state fMRI						
Levakov et al. [129]	Prognostic: Bodyweight	92 OW (WL: 29 PE, 29 PE + Mediterranean diet, 34 PE + polyphenol enriched Mediterranean diet)	T0 (pre-WL), T6 (after 6 months WL)	RS FC between atlas regions	OLS	RS_fMRI_T0 → OUT_T0_6: The authors detected a network primarily composed of reward regions for which the FC between more regions was related to WL on a suprathreshold level than was expectable by chance
Mokhtari et al. [132]	Prognostic: Bodyweight	52 OW/OB (WL & WM: caloric restriction, caloric restriction + aerobic exercise, caloric restriction + resistance exercise; no group sizes provided)	T0 (pre-WL), T18 (after 12-month WM + 6 months WL)	Sliding window RS FC between atlas regions	SVC (CV)	RS_fMRI_T0 → OUT_T0_18: Accuracy for classifying successful vs. non-successful WL based on sliding window RS FC of 97%
Contreras-Rodríguez et al. [125]	Prognostic: Bodyweight	42 OW/OB (WL: 30-min diet counseling session)	T0 (pre-WL), T3 (after 12 weeks WL)	Seed-to-voxel RS FC	OLS	RS_fMRI_T0 → OUT_T0_3: Negative Association between WL and RS FC between dorsal caudate and somatosensory cortex
McFadden et al. [124]	Associations: Bodyweight	11 OW/OB (WL: PE)	T0 (pre-WL), T6 (after 6 months WL)	Voxel-correlates of ICs extracted from RS fMRI	OLS	RS_fMRI_T0_6 → OUT_T0_6: Positive association between exercise-induced reduction in fat mass and exercise-induced reduction of default-mode network RS FC in precuneus
Structural neuroimaging						
Honea et al. [140]	Prognostic: Bodyweight	72 OB (WL: 72 cal restriction, physical exercise, behavioral modification)	T0 (pre-WL), T3 (after 12 weeks WL)	Voxel GM volume; voxel WM volume	OLS	VBM_sMRI_T0 → OUT_T0_3: Positive association between WL and GM volume in right parahippocampal gyrus and right OFC; VBM_sMRI_T0 → OUT_T0_3: Positive association between WL and WM volume close to left OFC/inferior frontal gyrus and right fusiform gyrus
Mokhtari et al. [141]	Prognostic: Bodyweight	52 OW/OB (WL & WM: 14 caloric restriction, 15 caloric restriction + aerobic exercise, 23 caloric restriction + resistance exercise)	T0 (pre-WL), T18 (after 12 months WMT + WL)	Voxel GM volume; voxel WM volume	SVC (CV)	VBM_sMRI_T0 → OUT_T0_18: Accuracy for classifying successful vs. non-successful WL based on: voxel-wise GM volume of 77% / WM volume 74% /GM & WM combined 78%
Best et al. [138]	Prognostic: Program adherence	Data set 1, 83 average waist-to-hip-ratio: 0.83 (WL: 33 once-weekly resistance training, 26 twice-weekly resistance training, 25 twice-weekly balance-and-tone training); Data set 2, 39 average waist-to-hip-ratio: 0.86 (WL: 13 resistance training, 14 aerobic training, 12 balance-and-tone training)	T0 (pre-WL) and T13 (Dataset 1: after 52 weeks WL) or T6 (Dataset 2: after 26 weeks WL)	Volume of atlas GM regions	LMMr	sMRI_sMRI_T0 → OUT_T0_13 and sMRI_T0 → OUT_T0_6.5: Across both data sets positive association between program attendance and GM volume of lateral OFC and middle frontal gyrus (i.e., dlPFC)
Gujral et al. [139]	Prognostic: Program adherence	159 (no group sizes and weight-related parameters provided): Moderate-intensity aerobic walking, nonaerobic stretching and toning condition	T0 (pre-PE program), T12 (after 12 months PE program)	Voxel GM volume; voxel FA	OLS	VBM_sMRI_T0 → OUT_T0_12: Positive association between program adherence and GM volume of (primarily) prefrontal, somatosensory, motor, temporal, and parietal regions; DTI_sMRI_T0 → OUT_T0_12: Positive association between program adherence and tract FA of (primarily) anterior thalamic radiation, forceps minor, superior longitudinal fasciculus, and body of the corpus callosum
Mueller et al. [143]	Associations: Bodyweight	16 OW/OB (WL: 60-min of supervised physical training twice a week)	T0 (pre-WL), T3 (after 3 months WL)	Voxel GM volume	OLS	VBM_sMRI_T0_3 → OUT_T0_3: Positive association between BMI reduction and GM volume in right insula and in left cerebellar regions

*ACC* anterior cingulate cortex, *ad lib* ad libitum, *BMI* body mass index, *CR* cue reactivity, *CV* cross validation, *DD* delay discounting, *dlPFC* dorsolateral prefrontal cortex, *dmPFC* dorsomedial prefrontal cortex, *FA* fractional anisotropy, *FC* functional connectivity, *fMRI* functional magnetic resonance imaging, *FUP* follow-up, *GLP1* Glucagon-like Peptide 1, *GM* grey matter, *LMM(r)* (robust) linear mixed model regression, *ncl* nucleus, *OB* obese, *OFC* orbitofrontal cortex, *OLS* ordinary least square regression, *OUT* outcome marker, *OW* overweight, *PE* physical exercise, *PFC* prefrontal cortex, *Pred. meth* computational prediction method, *RS* resting-state, *SEM* structural equation modelling, *sMRI* structural magnetic resonance imaging, *SVC* support vector classification, *VBM* voxel based morphometry, *vmPFC* ventromedial prefrontal cortex, *WL* weight loss, *WM* white matter, *WMT* weight maintenance

#### Delay discounting

DD tasks are well established experimental designs for the study of goal-directed control in obesity (e.g., [18, 97, 103–106]) in which participants have to decide multiple times between immediately available smaller rewards and larger delayed ones. Several methods exist for computing participant-specific voxel contrast maps reflecting goal-directed control in DD tasks such as contrasting more immediate options to more delayed ones [87], or contrasting difficult (similar attractiveness of immediate and delayed choices) vs. easy trials (dissimilar attractiveness; e.g., [106]). Another method is to first determine a behavioral measure of goal-directed control that allows modelling the subject-specific value of options (rewards) based on their reward magnitude and delay and to then compute the voxel-wise association between this model function and local activity (e.g., [97, 107]).

#### Food craving regulation

Paradigms requiring their participants to actively regulate affective states induced by generic emotional stimuli have either been applied without changes in obesity research or were slightly varied to study craving regulation induced by food stimuli. For example, a study investigating emotion regulation during presentation of generic affective stimuli found that obese persons have more emotion regulation difficulties assessed via questionnaires than controls. In addition, higher vmPFC activity during regulation is associated with less regulation difficulties [108]. Food CrvR paradigms (used for treatment outcome prediction in [68]) evaluate the effect of regulation strategies on food-cue elicited craving. Trials typically start by presenting a strategy word (e.g., “permit” or “regulate” [69]), which is followed by a high- or low-calorie food picture that should either be perceived in a permissive fashion (i.e., allowing oneself to perceive the potentially induced craving) or during application of a regulation strategy. Finally, participants rate their desire to consume the depicted food. Contrasting signals emerging during high-calorie food & permit (high-calorie food & regulate) vs. low-calorie food & permit (high-calorie food & permit) enables computing voxel activity maps for incentive salience (goal-directed control). Thus, this paradigm might be seen as a mixture of an incentive salience and goal-directed control task.

### Resting-state fMRI

RS fMRI measures spontaneous low-frequency brain activity under task-free conditions and has revealed fundamental aspects of how the brain is organized and works, i.e., its intrinsic organization in separate networks (i.e., the RS networks [109]) or that RS network activity impacts task-related activity of RS network [110].

#### Amplitude of low frequency fluctuations

The Amplitude of low frequency fluctuations (ALFF) method is applied in three reviewed BS studies [111–113] and characterizes spontaneous low-frequency brain activity by estimating the magnitude of these fluctuations in a small frequency band (e.g., from 0.01 to 0.08 Hertz [114]) for each voxel coordinate. Initially, the average square root of the power in this frequency band of a given voxel’s time series divided (i.e., standardized) by the average of this parameter across all voxels was used as voxel ALFF measure [115]. The improved fractional ALFF method uses the square root averaged across the full power spectrum for a given voxel as a standardization method [116]. It was suggested that frequency sub-bands within 0.01 to 0.08 Hertz reflect spontaneous low-frequency activity of different neural tissue types and that several diseases other than obesity induce alterations in ALFF (see [117] for an overview).

#### Functional connectivity density mapping

Functional connectivity density (FCD) mapping (employed in one reviewed study [118]), estimates the degree of FC for each voxel, and primarily aims to reveal areas of dense local FC (so-called “hubs”; [119]). Specifically, “local FCD” reflects the number of voxels in a cluster surrounding a center voxel having at least a predefined FC. “Global FCD” corresponds to the number of voxels having a suprathreshold FC with the center voxel irrespective of neighborhood minus its local FCD. The clinical relevance of FCD was e.g. supported by findings of an altered local FCD in schizophrenia [120] and a relation of FCD and severity of subclinical depressive symptoms in healthy elderly [121].

#### Independent component analysis

Independent component analysis is a statistical method that identifies RS networks by computing so-called independent components (ICs). These are transformations of the multivariate voxel input data which are stochastically independent (and not only mutually uncorrelated as in principal component analysis) and can be understood as characteristic RS voxel time series (e.g., [122]). One IC reflects the activity time course of one RS network. After the ICs are identified, they can either be related to treatment outcome directly as in [123] or relations between ICs/RS networks of interest and voxel-wise RS fMRI time courses are determined using participant-specific voxel-wise regression analysis. The resulting correlation/regression coefficients are finally handed over for a group analysis [124].

#### Seed-to-voxel functional connectivity

The seed-to-voxel FC for RS fMRI (“Seed-to-voxel RS FC”) applied by [125–127] is technically identical to its task-related counterpart described above.

#### Functional connectivity between anatomical atlas regions

FC for prediction has also been computed based on time series averaged across voxels located in anatomical atlas regions [111, 128–132]. Using averaged regional time series requires less priori knowledge as one can simply compute the FC between all atlas regions. Another advantage might be the method’s relative robustness to outlier voxels through spatial averaging. However, the method does not make full use of the spatial resolution fMRI is offering.

#### Functional connectivity network-analysis

This (group of) technique(s) aims at characterizing the structure of connected units interacting in complex social, economic, genetic, or neural networks [133]. One major aim is to assess the efficiency of network information flow [134]. Independent of the domain (e.g., social or biological) these techniques have shown that networks with a “small-world” structure (i.e., having a dense local and a sparse long-range connectivity) are both globally and locally efficient with regard to information flow because the average distance between any pair of units (here: brain regions) in such a network is small [135]. One of the reviewed studies [101] utilized a network technique for treatment outcome prediction. Specifically, these authors computed FC using the technique described in 3.2.5 for individual participants first and then determined the global efficiency (see [134]) for each participant-specific FC pattern for prediction.

### Neurotransmission assessed with Positron-Emission-Tomography

Positron-Emission-Tomography (PET) is a technique allowing to measure biochemical and physiological activity across biological tissues on a voxel-level by applying radio-tracers (e.g., [^11^C] raclopride and [^11^C] carfentanil) (employed in two reviewed studies [72, 73]). This method can be used in a task-related or RS fashion and has been applied extensively in obesity research to measure transmission of DA and other neurotransmitters (e.g., [15, 57–60]). Steele et al. related Roux-en-Y gastric bypass (RYGB)-induced D2R availability changes to weight loss in a 6-week period after surgery and report a positive association [72]. Karlsson et al. related presurgical μ-opioid receptor and D2R availability to post-BS weight [73]. While no associations were found for D2R, especially amygdala μ-opioid receptor availability was negatively associated to future body weight.

### Structural neuroimaging

#### Brain tissue volume

One of the most frequently evaluated tissue properties in structural neuroimaging in general and in the reviewed structural studies specifically [136–143] is voxel-wise tissue volume. Except for Best et al. [138], Voxel-Based Morphometry (VBM; [144]) was used in these studies for computation. VBM is implemented in SPM12 (Wellcome Trust Centre for Neuroimaging, Institute of Neurology, UCL, London UK ­ http://www.fil.ion.ucl.ac.uk/spm). In VBM, anatomical brain images are spatially registered to an anatomical reference space and segmented into the three tissue types grey matter (GM), white matter (WM), and cerebrospinal fluid (CSF). By additionally considering the amount of local deformation applied during spatial registration, the method produces markers of the voxel-wise volume for each of the three tissue types (referred to as “Voxel GM or WM volume” in the following). Contrary to VBM, the method used by Best et al. [138] computes volumes for larger regions included in an anatomical atlas and is implemented in FreeSurfer [145, 146]. For a method comparison, see Guo et al. [147].

#### Cortical thickness

Another structural brain property evaluated frequently in structural neuroimaging in general and in one reviewed studies [148] is cortical thickness (implemented e.g., in FreeSurfer [146]). In short, this parameter is computed by determining the WM/GM transition surface and the GM/pial transition surface in a first step and by determining the distance between these two surfaces for small spatial units (“vertices”) in a second.

#### Brain diffusion

Diffusion MRI enables evaluating brain fiber characteristics by assessing directions of water molecule diffusion and was applied in two of the reviewed studies [99, 139]. Specifically, utilizing the fact that water can diffuse equally into any direction in unstructured spaces such as CSF but only in directions predetermined by biological structures and their integrity in neural tissues, measurement and modelling of water molecule diffusion allows evaluating axon bundle orientation and integrity [149]. A method frequently used for this purpose is Diffusion Tensor Imaging (DTI). In DTI, water molecule diffusion is measured for a predefined number of diffusion orientations. Subsequently, participant- and time point-specific voxel maps reflecting different diffusion properties are determined by fitting an ellipsoid to the three-dimensional diffusion information. The two most important fiber characteristics derived thereof are Fractional Anisotropy, which can be understood as the degree of diffusion directedness, and Mean Diffusivity, a measure of overall diffusivity. Finally, a method complementing DTI is fiber tractography which aims at tracing WM tracts based on the directional information provided by diffusion-weighted MRI. One of the reviewed studies applied tractography [99]. Soares et al. provides an overview on DTI and tractography including a list of available software packages [150], Maier-Hein et al. highlights pitfalls in tractography [151].

## Computational prediction approaches

**Fig. 2:**
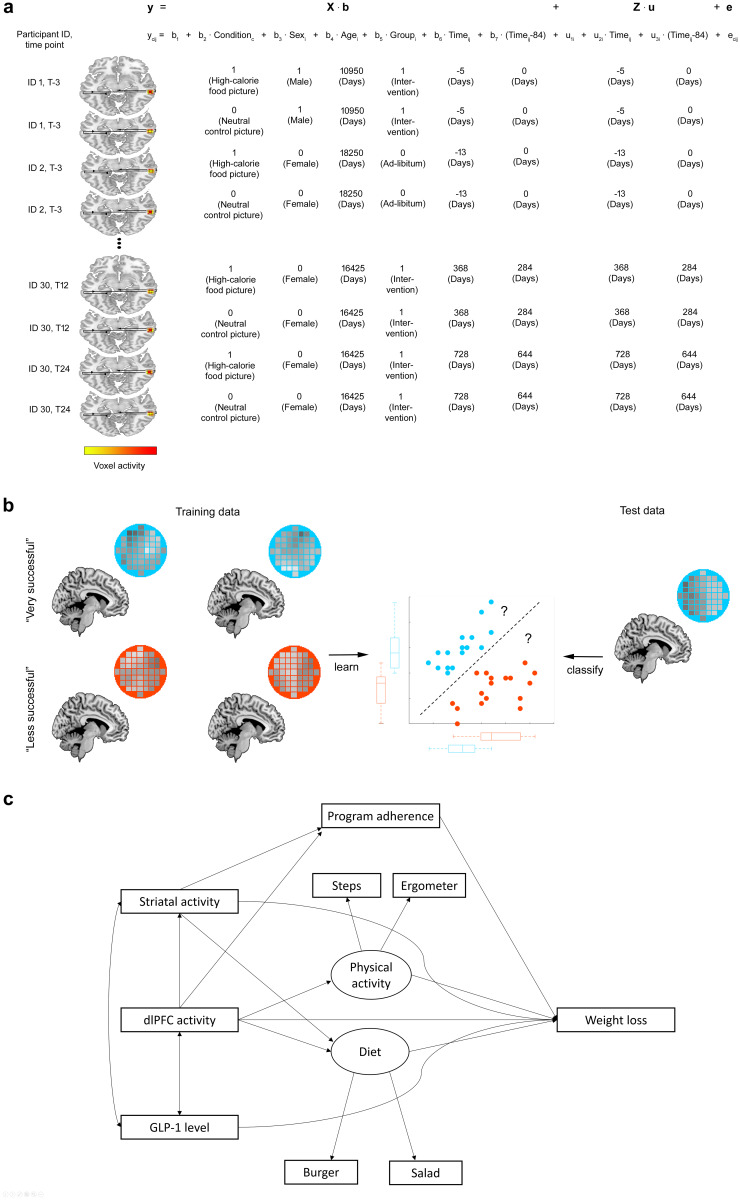
illustrates computational approaches used for treatment outcome prediction on the group level. In particular, (**a**) illustrates the LMM regression approach and is taken from [18]. (**b**) depicts an application of support vector classification for a hypothetical classification task in which a classifier has to learn the differences between voxel GM patterns belonging to very successful dieters and less successful dieters in the training stage. In the next step, the classification boundary estimated from the training data is used to predict the class of an unknown test person based on their GM pattern. (**b**) is derived from Weygandt et al. [172]. Finally, (**c**) shows a hypothetical structural equation model (in part derived from [169])

This section describes the methods used for assessing treatment outcome on the group level across the reviewed studies. Except for ordinary least squares (OLS) regression, these methods are illustrated in Fig. 2.

### Linear regression

#### Ordinary least square regression

The technique used for treatment outcome prediction on the group level in the majority of reviewed studies is voxel-wise OLS regression. This technique is implemented in a variety of software packages such as SPM, FMRIB Software Library (FSL) [152], Analysis of Functional NeuroImages (AFNI; http://afni.nimh.nih.gov/afni) [153], or BrainVoyager (http://www.brainvoyager.com/) [154]. OLS regression identifies an optimal set of regression coefficients by minimizing the sum of squared differences between true and predicted values for the dependent variable (e.g., weight loss obtained in a certain interval or brain activity in a certain voxel). Given that multiple voxels are tested, methods correcting for family-wise error (e.g., the Random Field Theory, Bonferroni, or the maximum statistic method [155–157]) have to be applied to evaluate the significance of individual voxels’ tests. A drawback in a longitudinal framework is the method’s sensitivity to drop-out, as participants have to be excluded completely once a single time point is missing (for further points, see e.g. [158, 159]).

#### Linear mixed model regression

Linear mixed models (LMMs; implemented in Freesurfer [160, 161] are a newer regression method [162] that has been applied in four reviewed studies [18, 136, 138, 163]. LMM regression models the variation in the criterion (e.g., weight loss) as a linear combination of fixed and random effects. The former correspond to parameters that can be defined freely by the researcher (e.g. group membership), the latter to parameters for which this is not possible because they vary in an endogenous, participant-specific fashion (e.g., participants’ signal means or trends). LMM regression has several properties that makes it a suitable candidate for the analysis of longitudinal study designs. First, it can model a signal sampled across an arbitrary number of time points simultaneously. Second, it can handle designs which are unbalanced due to participant drop out [162] and has thus has higher statistical power than alternative methods (e.g., repeated measures analysis of variance based on OLS regression) because participants with partially missing data need not to be excluded [161]. It has been shown that even the inclusion of participants with only a single data point can improve the accuracy of LMM regression [161]. Finally, unlike repeated measures analysis of variance, LMM regression is able to cope flexibly with varying data covariation across time.

### Support vector classification

Support vector classification (SVC) is a supervised classification approach used in the majority of reviewed studies employing machine learning (i.e., [132, 141]). A third study employed a combination of Twin networks and k-nearest neighbor clustering [130]. Given the rare use of this approach in neuroimaging, we would like to point the reader directly to this study for details. In supervised classification, a machine learning algorithm tries to learn characteristic properties from (e.g., brain activity) patterns representative of different classes (e.g., successful vs. non-successful dieters) in a training stage. In its basic form, the SVC algorithm does so by identifying a linear class boundary (“classification model”) that separates the training patterns of two classes and which optimizes the trade-off between the number of non-separable patterns and classifier complexity [164]. In the test or model validation stage, the model is evaluated by computing a classification accuracy measure for unseen test data. Model validation techniques range from leave-one-out cross validation (LOO-CV) to out-of-sample validation. One important aspect arising from this variety and other factors such as a putative sensitivity of supervised classification to sample size [165] is the use of resampling techniques for inference. These consider the conditions under which the empirical classification accuracy is obtained. Parametric procedures do not possess this property (e.g., [166, 167]).

### Structural equation modelling

Structural equation modelling [168] is a multivariate analysis technique applied in one reviewed study [169] evaluating whether/how well the relations among hypothesized constructs fit to relations among (“latent” or unobservable) mathematical factors representing these constructs extracted from a set of (“manifest” or observable) empirical data. In structural equation modelling, relations between manifest variables and latent constructs (i.e., the “measurement model”) and among latent constructs (“latent variable model”) have to be specified first. It is possible to specify directed effects in the latent variables model and thus to assume causal relations among variables (see below). Although structural equation modelling is a very flexible and powerful tool, several aspects have to be taken into consideration. Structural equation modelling cannot be used to test causal relations among constructs. Instead, structural equation modelling tests whether associations in a specific empirical data set fit to the causal assumptions held by the researcher. Poor model fits will strongly question the validity of these assumptions. However, good model fits will increase their plausibility, but not prove them, and require replication on independent data sets [170]. The sensitivity of structural equation modelling to variations in sample size and to violations of distributional assumptions remains a limiting factor. Methods to deal with these problems have e.g., been presented by Kock & Hadaya [171] and Hox et al. [172]. See [172] for an overview.

## Study overview

Here we provide a tabular overview of the reviewed studies. Table 1 gives an overview on LI-based studies, Table 2 on BS-based studies and Table 3 on PI-based studies.

**Table 2:** Overview on existing bariatric surgery studies. Studies are subdivided by neuroimaging technique. Studies may be listed more than once if more than one neuroimaging technique is applied. For the interpretation of parameters reported in columns “Modelling: Outcome”, “Predictor”, and “Significant prediction results”, please see Table 1

Study	Modelling: Outcome	#Participants (per group)	Experimental design	Predictor	Pred. meth	Significant prediction results
Task-fMRI						
Bach et al. [85]	Prognostic: Bodyweight	11 OB: 10 RYGB, 1 SG	T0 (2 weeks before surgery) T6.5 (24 weeks after surgery)	Voxel CR activity	OLS	CR_fMRI_T0 → OUT_T0_6.5: No association between WL and evaluated neuroimaging predictors was found
Smith et al. [176]	Prognostic: Bodyweight	39 OB: 19 RYGB, 20 SG	T0 (pre surgery), T6 (after 6 months)	Voxel CR activity	OLS	CR_fMRI_T0 → OUT_T0_6: Negative association between WL and CR activity of ventral tegmental area after RYGB (but not SG)
Holsen et al. [196]	Prognostic: Bodyweight	18 OB: SG	T0 (pre surgery), T12 (12 months after surgery)	Voxel CrvR activity	OLS	CrvR_fMRI_T0 → OUT_T0_12: Negative association between WL and upregulated CR activity in ncl. accumbens and hypothalamus
Ness et al. [178]	Prognostic: Bodyweight	19 OB: LAGB	T0 (pre surgery), T3 (3 months after surgery), T6 (6 months after surgery)	Voxel CR activity	OLS	CR_fMRI_T0 → OUT_T0_3: Positive association between WL and CR activity in left superior frontal and transverse temporal gyrus; Negative association between WL and CR activity in right and left post. cerebellar lobe; CR_fMRI_T0 → OUT_T0_6: Positive association between WL and CR activity in right inferior temporal and frontal gyrus, right occipital cortex, left and right post. cerebellar lobe; Negative association between WL and CR activity in right cuneus, left precuneus, and left superior frontal gyrus
Bruce et al. [197]	Prognostic: Eating behavior	10 OB: LAGB	T0 (pre surgery), T3 (12 weeks after surgery)	Voxel CR activity	OLS	CR_fMRI_T0 → OUT_T0_3: Negative association between reduction in disinhibited eating behavior and CR activity in right inferior frontal gyrus
Hu et al. [99]	Associations: Bodyweight	25 OB: SG	T0 (pre surgery), T1 (1 month after surgery), T6 (6 months after surgery)	Seed-to-voxel CR FC; averaged voxel FA in tracts connecting atlas regions	OLS	CR_fMRI_T0_1 → OUT_T0_1: Positive association between WL and changes in CR FC between right dlPFC and ACC; DTI_sMRI_T0_6 → OUT_T0_6: Positive association between WL and changes of average FA of tract connecting right dlPFC and pregenual ACC
Li et al. [100]	Associations: Bodyweight	22 OB: SG	T0 (pre surgery), T1 (1 month after surgery)	Seed-to-voxel CR FC	OLS	CR_fMRI_T0_1 → OUT_T0_1: Positive association between surgery-induced WL and surgery-induced increase in CR FC of right dlPFC and ACC
Zoon et al. [198]	Associations: Eating behavior	19 OB: RYGB	T0 (pre surgery), T2 (2 months after surgery)	Voxel CR activity	OLS	CR_fMRI_T0_2 → OUT_T0_2: Negative association between surgery-related shift in food preferences from high-fat/sweet to low-energy/savory foods and surgery-related CR activity of superior parietal lobule in response to high-energy food odor and with surgery-related activity of precuneus in response to high-energy food pictures
Ochner et al. [177]	Associations: Food craving	14 OB: RYGB	T0 (1 month before surgery), T2 (1 month after surgery)	Voxel CR activity	OLS	CR_fMRI_T0_2 → OUT_T0_2: Positive association between surgery-induced changes in food wanting and changes in CR activity of caudate, lentiform nucleus, middle and superior frontal gyri, inferior parietal lobule, anterior cingulate, and thalamus. Positive association between surgery-induced changes in food liking and changes in precuneus CR activity
Resting-state fMRI						
Zhang et al. [130]	Prognostic: Bodyweight	37 OB: SG	T0 (pre surgery), T6 (after 6 months)	Principal components of RS FC between atlas regions	TWK (CV)	RS_fMRI_T0 → OUT_T0_18: Accuracy for classifying successful vs. non-successful WL based on RS FC of 84%
Cerit et al. [126]	Prognostic: Bodyweight	14 OB: SG	T0 (1 month before surgery), T13 (12 months after surgery)	Seed-to-voxel RS FC	OLS	RS_fMRI_T0 → OUT_T0_13: Positive association between WL and RS FC between right ncl. accumbens and left insula and between WL and RS FC between left hypothalamus and left precentral gyrus
Olivo et al. [131]	Prognostic: Bodyweight	16 OB: RYGB	T0 (1 month before surgery) T2 (1 month after surgery), T13 (12 months after surgery)	RS FC between atlas regions	OLS	RS_fMRI_T0 → OUT_T0_13: Positive association between WL and RS FC of right paracingulate gyrus and the right amygdala
Schmidt et al. [127]	Associations: Bodyweight	14 OB: RYGB	T0 (pre surgery), T8 (8 months after surgery)	Seed-to-voxel RS FC	OLS (CV)	RS_fMRI_T0_8 → OUT_T0_8: Association between surgery-induced body weight changes and RS FC of ventromedial PFC and ventral striatum
Duan et al. [112]	Associations: Bodyweight	16 OB: SG	T0 (pre surgery), T1 (1 month after surgery)	Voxel ALFF	OLS	RS_fMRI_T0_1 → OUT_T0_1: Positive association between surgery-induced WL and surgery-induced ALFF reduction in OFC
Li et al. [111]	Associations: Bodyweight	22 OB: SG	T0 (pre surgery), T1 (1 month after surgery)	Voxel FCD	OLS	RS_fMRI_T0_1 → OUT_T0_1: Negative association between surgery-induced WL and surgery-induced change in local FCD in vmPFC and posterior cingulate gyrus/precuneus
Li et al. [118]	Associations: Bodyweight	17 OB: SG	T0 (pre surgery), T4 (4 months after surgery)	Voxel fractional ALFF; RS FC between atlas regions	OLS	RS_fMRI_T0_4 → OUT_T0_4: Association between body weight and fractional ALFF in reward processing and cognitive control regions comprising OFC, gyrus rectus, superior frontal gyrus, and middle frontal gyrus RS_fMRI_T0_4 → OUT_T0_4: Association between body weight and FC between reward processing and cognitive control regions comprising OFC, gyrus rectus, superior frontal gyrus, and middle frontal gyrus
Dong et al. [128]	Associations: Addiction- like symptoms	14 OB: SG	T0 (pre surgery), T6 (6 months after surgery)	RS FC between atlas regions	OLS	RS_fMRI_T0_6 → OUT_T0_6: Positive association between addiction-like obesity symptoms (e.g., continued use despite problems, tolerance, withdrawal, dependence on food) and RS FC of precuneus and putamen
Zhang et al. [113]	Associations: Anxiety	30 OB: SG	T0 (pre surgery), T1 (1 month after surgery)	Voxel ALFF	OLS	RS_fMRI_T0 _1 → OUT_T0_1: Positive association between surgery-induced WL and surgery-induced ALFF reduction in OFC
Saindane et al. [123]	Associations: Cognitive functions	8 OB: Type of surgery not described	T0 (3 to 0 months before surgery), T4.5 (0 to 6 months after surgery)	ICs extracted from RS fMRI	OLS	RS_fMRI_T0_9 → OUT_T0_9: Positive association between surgery-related changes in pattern comparison performance and surgery-related changes in RS FC of a left executive control network
Positron-Emission-Tomography						
Karlsson et al. [73]	Prognostic: Bodyweight	19 OB: 6 RYGB, 13 SG	T0 (pre surgery), T3 (3 months after surgery), T6 (6 months after surgery), T12 (12 months after surgery), T24 (24 months after surgery)	Voxel [^11^C] carfentanil radiotracer binding; voxel [^11^C]raclopride binding	OLS	PET_T0 → OUT_T3: Negative association between body weight and μ-opioid receptor availability in amygdala, insula, ventral striatum, and putamen PET_T0 → OUT_T6 Negative association between body weight and μ-opioid receptor availability in amygdala PET_T0 → OUT_T12 Negative association between body weight and μ-opioid receptor availability in amygdala PET_T0 → OUT_T24 Negative association between body weight and μ-opioid receptor availability in amygdala and thalamus PET_T0 → OUT_T3, PET_T0 → OUT_T6, PET_T0 → OUT_T12, PET_T0 → OUT_T24: For none of the time points, an association between future body weight and D2R availability was found
Steele et al. [72]	Associations: Bodyweight	5 OB: RYGB	T0 (Pre surgery), T1.5 (4 to 6 weeks after surgery)	Voxel [11C] raclopride radiotracer binding	VIS	PET_T0_1.5 → OUT_T0_1.5: Negative association between surgery-related changes in bodyweight and surgery-related changes in D2-receptor availability
Structural neuroimaging						
Tuulari et al. [142]	Prognostic: Bodyweight	40 OB: 19 RYGB, 21 SG	T0 (pre surgery), T6 (6 months after surgery)	Voxel GM volume; voxel WM volume	OLS	VBM_sMRI_T0 → OUT_T0_6: Positive associations between WL and GM volume in frontotemporal, thalamic, insular, and cerebellar areas; VBM_sMRI_T0 → OUT_T0_6: Positive association between WL and WM volume in frontotemporal areas
Hu et al. [99]	Associations: Bodyweight	25 OB: SG	T0 (pre surgery), T1 (1 month after surgery), T6 (6 months after surgery)	Averaged voxel FA in tracts connecting atlas regions; Seed-to-voxel CR FC	OLS	DTI_sMRI_T0_6 → OUT_T0_6: Positive association between WL and changes of average FA of tract connecting right dlPFC and pregenual ACC; CR_fMRI_T0_1 → OUT_T0_1: Positive association between WL and changes in CR FC between right dlPFC and ACC
Michaud et al. [136]	Associations: Bodyweight	29 OB: SG	T0 (1 month before surgery), T5 (4 months after surgery), T13 (12 months after surgery)	Voxel WM volume averaged across atlas regions	LMM	VBM_sMRI_T0_T5_T13 → OUT_T0_T5_T13: Negative association between body weight variation and WM volume in the cingulum, cerebellar peduncle, and parietopontine tract
Liu et al. [148]	Associations: Bodyweight	22 OB: SG	T0 (pre surgery), T1 (1 month after surgery)	CT aggregated across vertices in atlas regions	OLS	CT_sMRI_T0_1 → OUT_T0_1: Uncorrected negative association between WL and CT of the superior frontal gyrus
Rullmann et al. [137]	Associations: Bodyweight	27 OB: RYGB	T0 (pre surgery), T6 (6 month after surgery), T12 (12 month after surgery)	Voxel GM volume	OLS	VBM_sMRI_T0_6 → OUT_T0_6: Negative association between WL and GM volume in the hypothalamus and the left postcentral gyrus VBM_sMRI_T0_6 → OUT_T0_6: Positive association between WL and GM volume in the right lateral OFC

*ACC* anterior cingulate cortex, *ALFF* Amplitude of low frequency fluctuations, *CrvR* craving regulation, *CT* cortical thickness, *CR* cue reactivity, *CV* cross validation, *dlPFC* dorsolateral prefrontal cortex, *dmPFC* dorsomedial prefrontal cortex, *FA* fractional anisotropy, *FC* functional connectivity, *FCD* functional connectivity density, *fMRI* – functional magnetic resonance imaging, *GM* grey matter, *IC* independent components, *LAGB* laparoscopic adjustable gastric banding, *LMM(r)* (robust) linear mixed model regression, *ncl* nucleus, *OB obese OFC* orbitofrontal cortex, *OLS* ordinary least square regression, *OUT* outcome marker, *OW* overweight, *PET* Positron-Emission-Tomography, *PFC* prefrontal cortex, *Pred. meth* Computational prediction method, *RS* resting-state, *RYGB* Roux-en-Y gastric bypass, *SG* sleeve gastrectomy, *sMRI* structural magnetic resonance imaging, *TWK* twin networks and k-nearest neighbor clustering, *VBM* voxel based morphometry, *VIS* visual interpretation, *vmPFC* ventromedial prefrontal cortex, *WL* weight loss, *WM* white matter

**Table 3:** Overview on existing pharmacological intervention studies. For the interpretation of parameters reported in columns “Modelling: Outcome”, “Predictor”, and “Significant prediction results”, please see Table 1

Study	Modelling Outcome	#Participants (per group)	Experimental design	Predictor	Pred. meth	Significant prediction results
Task-fMRI						
Ten Kulve et al. [86]	Prognostic: Bodyweight	20 OW/OB: Liraglutide (vs. Insulin; cross-over design)	T0 (before treatment), T0.3 (after 10 days of treatment), T3 (after 12 weeks of treatment)	Voxel CR activity	OLS	CR_fMRI_T0.3_3 → OUT_T0_12: No association between WL and evaluated neuroimaging predictors was found
Ten Kulve et al. [199]	Prognostic: Bodyweight	20 OW/OB: Liraglutide (vs. Insulin; cross-over design)	T0 (before treatment), T0.3 (after 10 days of treatment), T3 (after 12 weeks of treatment)	Voxel CR activity	OLS	CR_fMRI_T0.3 → OUT_T0_12: Positive association between WL and higher CR activity in right insula after liraglutide vs. after insulin

*CR* cue reactivity, *fMRI* functional magnetic resonance imaging, *OB* obese, *OLS* ordinary least square regression, *OUT* outcome marker, *OW* overweight, *Pred. meth* computational prediction method, *WL* weight loss

## Discussion

In this study, we review current work on computational approaches to predicting treatment response in obesity using neuroimaging. We started by outlining key CNS mechanisms thought to affect treatment outcome and then described the neuroimaging techniques and parameters as well as computational approaches used for prediction. Lastly, we gave an overview on existing studies.

This overview provided a consistent picture on the role of CNS mechanisms for treatment outcome in obesity. For example, the importance of dopaminergic reward areas was underlined by CR studies showing that cue-evoked activity in these areas is negatively related to treatment outcome [173–177]. The relevance of goal-directed control regions comprising frontal and parietal areas as well as insula was demonstrated in DD tasks directly designed to study goal-directed control [18, 97, 104, 106] and in CR tasks by showing that cue-related activity of these areas has a positive effect on treatment outcome [86, 169, 178–180]. Task-derived FC and RS FC studies showed that higher FC between fronto-parietal and insular goal-directed control areas on one hand and incentive salience areas on the other is accompanied by better treatment outcomes [18, 97, 99, 100, 126]. Obesity-related regions involved in incentive salience and goal-directed control as identified by task fMRI strongly overlap with obesity-related regions as identified via RS FC [181]. These results were complemented by structural neuroimaging studies [99, 137–140, 142, 143] showing that higher GM volume of goal directed areas was associated with better treatment outcome and higher GM volume of incentive salience regions with worse outcome.

Some studies did not support a link between incentive salience or goal-directed control and treatment outcome. The fMRI studies of Bach et al. and Ten Kulve et al. did not show such a link [85, 86]. In addition, associations between cortical thickness of the superior frontal gyrus and weight loss reported by Liu et al. did not reach a multiple comparison corrected significance level [148]. Similarly, a couple of behavioral studies (not properly controlling for well-known nuisance factors) failed to identify reduced goal-directed control in obesity (e.g., [94, 95]). Thus, due to these null results and the possibility of a publication bias [182], the large number of consistent findings mentioned have to be viewed in a critical light. Nevertheless, given that the concepts of incentive salience and goal-directed control are derived from basic neuroscience on reward and motivation (e.g., [75, 76]), it can be assumed that these two CNS mechanisms play an important role for treatment outcome in obesity.

Do the presented findings also allow us to conclude that the evaluated neuroimaging predictors yield suitable biomarkers for obesity treatment outcome in a real-world precision medicine approach? Such a conclusion might be premature. An important counter argument is that the majority of studies applied correlational techniques evaluating all available data in one step (instead of applying model validation techniques) to analyze many predictors for one criterion assessed in small to moderately sized samples. This approach is sensitive to over- (and under-) fitting [183]. If the number of predictors is high (e.g., as in voxel-wise analyses) and the number of participants small, a statistical model for a criterion can occasionally fit this criterion well (poorly) because the predictor is not very reliable or varies substantially across different measurements. Thus, although such correlational analyses yield valid statistical inference on the group level if the analyses adequately controlled for multiple comparisons and were not circular [184, 185], their results might not be generalizable to unseen data and would not yield suitable biomarkers. Consequently, suitable biomarkers have to have a high retest-reliability.

How reliable are which neuroimaging parameters? What can be done to improve data reliability? How can a high generalizability of a prognostic model to unseen data be ensured? Meta-analyses provide answers to the first question. Studies assessing the reliability of task fMRI (typically via the intraclass correlation (ICC; [186]) for which a poor reliability was defined as ICC < 0.4, a fair as 0.4 ≤ ICC < 0.59, a good one as 0.6 ≤ ICC < 0.75, and an excellent as ICC ≥ 0.75 [187]) reported an average ICC of 0.5 ([188]; N = 15) or 0.397 ([189]; N = 56). A meta-analysis assessing retest-reliabilities for RS FC found an average ICC of 0.29 ([190]; N = 25). Consistent with Han et al. [191], retest-reliabilities computed for volume- and surface-based structural neuroimaging parameters by Elliott et al. showed primarily excellent ICCs [189]. Thus, together with the fact that structural neuroimaging parameters were significantly related to treatment outcome, these findings show that structural neuroimaging parameters might provide the most suitable biomarkers.

However, it is unclear to which degree the reliabilities reported for task fMRI and RS FC can be generalized. First, clinical studies applying specific tasks due to disease-related theoretical or empirical reasons were severely underrepresented in these meta-analyses. Second, especially for Elliott et al., the average retest interval was quite long (four months) given that Bennett & Miller found that studies with three or more months had reduced retest-reliability [188, 189]. The latter finding is consistent with the fact that retest-reliability is affected by a biomarker’s noise as well as by noise-independent physiological alterations occurring over time [192].

Irrespective of whether or not these meta-analyses provide extremely accurate estimates of retest-reliability for fMRI-derived markers, there is space for improvement. Consequently, one might ask what to do to improve reliability? Circumstances enabling an accurate reliability estimation are an important prerequisite. Given that retest-reliability is negatively associated with the duration of the retest-interval [192], an unbiased estimate requires a short time interval. A cross-sectional estimation procedure with zero interval length might be optimal in this regard which computes reliability based on several data subsets of a single scanning session [193]. Given that fMRI data reflect a highly complex process and are sensitive to a broad range of confounding factors, improvements should aim at adequately reducing factors such as head motion, breathing, heart rate, hydration, satiation, neuromodulators including caffeine or nicotine (e.g., [183]). Specific improvements for FC comprise increasing the number of acquired fMRI scans [189, 190] and combining RS FC data acquired across extended scan sessions with complementary task-fMRI data [189]. Additionally, FC computation based on a natural viewing tasks yielded fair to excellent reliability in a study of Wang et al. and was significantly higher than that derived from RS [194].

Another step to optimize neuroimaging-based treatment outcome prediction for clinical application would aim at maximizing the generalizability of a prognostic model to unseen data via application of model validation techniques. In this approach, different models (e.g., derived from neural networks, support vector classifiers, etc.) would be trained on a set of training data. Selection of features yielding a high prognostic accuracy could then be performed for each model separately using independent evaluation data. The prognostic performance is then tested once for each model based on independent test data and the best is selected for application (e.g., [183]).

Consequently, access to highly reliable biomarkers derived from adequately powered studies using model validation techniques is an important prerequisite for using neuroimaging-derived biomarkers for applied treatment outcome prediction in clinical practice – a prerequisite that might not be met today. Besides these application-related aspects, further improvements could entail a more elaborate modelling of “treatment-outcome”. This would take multiple neuroimaging, hormonal, and outcome markers (see e.g., [18, 163, 179]) into consideration. In this regard, the study of Szabo-Reed and colleagues might be pioneering as complex associations between brain activity, caloric restrictions, program attendance, physical activity and weight loss were modeled within a single structural equation model in this work [169]. This approach does not only promise to reveal a more fine-grained picture of contributing factors but also to facilitate a comparison of prediction accuracy obtained by different biomarker compilations.

In conclusion, the reviewed studies provide consistent support for the importance of incentive salience and goal-directed control as central nervous mechanisms mediating treatment outcome in obesity. Despite these findings, larger studies using statistical methods optimized with regard to real-world outcome prediction are needed to determine whether the approach is sufficiently accurate for application in a personalized medicine framework.

## Data Availability

Not applicable.

## Abbreviations

ALFF: Amplitude of low frequency fluctuations
BMI: Body mass index
BS: Bariatric surgery
CR: Cue reactivity
CrvR: Craving regulation
CSF: Cerebrospinal fluid
D2R: Dopamine receptor subtype D2
DA: Dopamine
DD: Delay discounting
dlPFC: Dorsolateral prefrontal cortex
DTI: Diffusion tensor imaging
FC: Functional connectivity
FCD: Functional connectivity density
fMRI: Functional magnetic resonance imaging
GM: Grey matter
IC: Independent component
LAGB: Laparoscopic adjustable gastric banding
LI: Lifestyle intervention
LMM: Linear mixed model
LOO-CV: Leave-one-out cross validation
MRI: Magnetic resonance imaging
OLS: Ordinary least squares
PE: Physical exercise
PET: Positron-Emission-Tomography
PI: Pharmacological intervention
RS: Resting-state
RYGB: Roux-en-Y gastric bypass
SG: Sleeve gastrectomy
sMRI: Structural magnetic resonance imaging
SVC: Support vector classification
VBM: Voxel-based morphometry
vmPFC: Ventromedial prefrontal cortex
WM: White matter
